# Photoreactive Capture and Conversion of Dilute Carbon Dioxide into Synthetic Natural Gas

**DOI:** 10.1021/acsaem.5c01559

**Published:** 2025-09-10

**Authors:** Sawyer Halingstad, Noemi Leick, Zhe Huang, James M. Crawford, Gerard Michael Carroll, Gabrielle A. Kliegle, James L. Young, Alexander J. Hill, Randy Cortright, Matthew M. Yung, Wade A. Braunecker

**Affiliations:** † 53405 National Renewable Energy Laboratory, Golden, Colorado 80401, United States; ‡ 33052 Montana State University, Bozeman, Montana 59717, United States

**Keywords:** Photoreactive carbon capture, Methane production, TiO_2_-based photocatalysis, Amine-functionalized sorbents, Plasmon-enhanced catalysis

## Abstract

This study introduces a photoreactive system that integrates the capture of dilute CO_2_ streams with their catalytic conversion to synthetic natural gas (CH_4_), utilizing a Ru nanoparticle (NP)-doped TiO_2_ composite loaded with linear polyethylenimine (L-PEI) and enhanced with plasmonic titanium nitride (TiN). This light-driven approach mitigates challenges that have plagued traditional thermal reactive carbon capture (RCC) methods, such as CO_2_ slip and amine degradation. We demonstrate that L-PEI enables stable CO_2_ capture and conversion, achieving ∼70% conversion of captured CO_2_ to CH_4_ across multiple reaction cycles using nonflammable forming gas (∼5% H_2_) as the reductant. In contrast, branched PEI (B-PEI)-loaded composites exhibited significant catalyst deactivation after several RCC cycles. Scanning transmission electron microscopy (STEM) imaging confirms that significant sintering of the Ru NPs occur in the B-PEI sample under RCC conditions, whereas their size remains stable in more rigid L-PEI composites. Technoeconomic analysis (TEA) estimates that CH_4_ production using this system could cost less than $5/kg based on current electrocatalytic H_2_ prices. These results represent one of the most promising demonstrations of amine-based RCC employing dilute CO_2_ sources to date.

## Graphical abstract

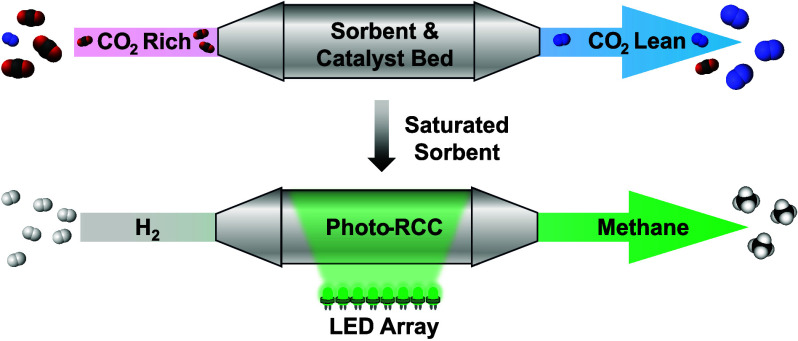

While the drive to develop more efficient carbon capture technologies has bolstered the environmental policy objectives of many nations,^1^ an often-overlooked aspect of these technologies is their potential to create new economic opportunities. Indeed, there is growing interest in market-driven concepts that utilize captured CO_2_ to produce value-added chemicals and fuel precursors.^2^ Among these concepts, reactive carbon capture (RCC)—which integrates sorbent-based CO_2_ capture and catalytic conversion within a single composite material and step—is a promising approach to enable process intensification and subsequent cost reduction of CO_2_ conversion processes.^3^

Certain carbon capture technologies have reached the pilot or precommercial stage.^4^ However, RCC research remains largely confined to the lab-scale. Recent perspectives highlight the opportunities and challenges in this emerging field, focusing on diverse sorbent/catalyst combinations and regeneration techniques that can improve technoeconomic assessments.^5,6^ Most thermal RCC systems using amines are designed for liquid-phase point-source CO_2_ capture processes, employing homo- or heterogeneous metal catalysts and relying on pressurized H_2_ swings (typically over 30 bar) to drive CO_2_ reduction.^5^ However, scaling RCC cost-effectively remains challenging due to mismatches between CO_2_ capture and conversion rates, amine stability under catalytic conditions, catalyst passivation during real-world operations, and energy intensive temperature, moisture, or pressure swings.^6^

In contrast to point-source capture, adapting RCC processes for dilute CO_2_ sources and negative emission technologies has proven particularly challenging due to the additional constraints specific to those systems. Efficiently removing CO_2_ with direct air capture (DAC) requires much larger volumes of air to contact the sorbent than many traditional liquid-phase systems can manage.^7^ In amine-based DAC, this requires using tethered small molecule amines or nonvolatile polymeric amines loaded on high surface area solid-state supports,^8^ which can complicate RCC integration since the captured CO_2_ may not attain the same intimate contact with the catalyst that it could in a liquid-phase system. Additionally, most amines release CO_2_ below 100 °C,^9^ far below the temperatures needed for efficient catalytic reduction—typically over 200 °C for methanation and well above 300 °C for the reverse water–gas shift.^10^ As a result, CO_2_ desorbs from the sorbent long before the necessary catalytic temperatures are reached in typical thermal swing systems, a phenomenon known as ″CO_2_ slip″.^9,11^ Further, amine sorbents degrade rapidly at the high temperatures required for these catalytic reactions, further limiting the approach’s viability.^12^ For these reasons, implementing thermal RCC in amine-based DAC systems remains largely impractical.

Photocatalysis has emerged as a promising strategy to address these challenges. Photoexcited electrons and holes can significantly lower the activation energies for certain CO_2_ reduction reactions.^13^ With the right catalyst, light energy can in principle drive these reactions at much lower bulk temperatures than traditional thermal processes, potentially reducing CO_2_ slip and mitigating the thermal degradation of amine sorbents. Moreover, photocatalytic processes are highly compatible with renewable and intermittent electricity sources, making them ideal for small, modular deployments. This flexibility makes photocatalysis particularly suited for remote locations where large-scale infrastructure or waste industrial heat required for many state-of-the-art capture systems is unavailable.

Numerous photocatalysts have been explored for the reduction of CO_2_ into synthetic fuels and chemical precursors.^14^ Titanium dioxide (TiO_2_) is particularly well-suited for this purpose owing to its dual role as a known photocatalyst^15^ and a high-surface-area mesoporous support commonly used in DAC applications.^16^ For example, ruthenium (Ru) loaded on TiO_2_ has long been recognized for its ability to catalyze photomethanation under milder conditions compared to thermal processes.^17^ In the decades since this discovery, variations in the support material and their influence on catalytic mechanisms have been extensively investigated.^18–20^ The incorporation of efficient visible light absorbers such as titanium nitride (TiN) has further enhanced photocatalytic methanation, with TiN’s plasmonic properties significantly improving light absorption and driving CO_2_ reduction under visible light.^21^

Recent advancements have demonstrated that tethering small molecule amines to TiO_2_ can create a dual-function photocatalytic system capable of both capturing and reducing CO_2_.^22^ Building on all these innovations, this study explores several amine-based photo-RCC systems, along with dilute CO_2_ sources and nonflammable forming gas (5% H_2_), to generate synthetic natural gas (CH_4_). Operating at ambient pressure and temperature, this approach enables reactive capture and conversion of dilute CO_2_ streams, offering a scalable and distributed solution for CO_2_ utilization within renewable energy frameworks.

Our materials preparation and activation procedures are detailed in the Supporting Information (SI), along with basic characterization including X-ray diffraction, nitrogen isotherms, thermogravimetric analysis, diffuse reflectance IR, and diffuse reflectance measurements with a UV–vis spectrometer (Figures S1–S5). Briefly, we first synthesized a TiO_2_ catalyst loaded with 5 wt % Ru nanoparticles (NPs), with and without an additional 5 wt % TiN. Initial photomethanation experiments using these catalytic materials (Figure 1) were conducted under steady-state conditions (10 sccm of 2000 ppm of CO_2_, 4% H_2_, balance N_2_). Results indicate that TiN significantly lowers the irradiance required to initiate photomethanation relative to the sample with only Ru (we further note that in the absence of Ru, no methanation was observed). For example, in the presence of TiN, >50% conversion of CO_2_ to CH_4_ is achieved with a green LED at an irradiance of 0.8 W/cm^2^ (30% of the LED’s maximum intensity), a yield unattainable even at 2.6 W/cm^2^ (100% intensity) without TiN. The green LED was chosen because its emission spectra overlapped well with the broad absorption feature of the composites observed in the diffuse reflectance measurements (Figures S5 and S6). A thermal imaging camera was used to monitor bulk sample temperature as a function of irradiance (Figures S9 and S10), revealing that at 2.0 W/cm^2^, the sample heated to ∼200 °C, the upper thermal stability limit for most amine systems.^23^ In subsequent reactions, we kept the irradiance below this intensity.

**Figure 1:**
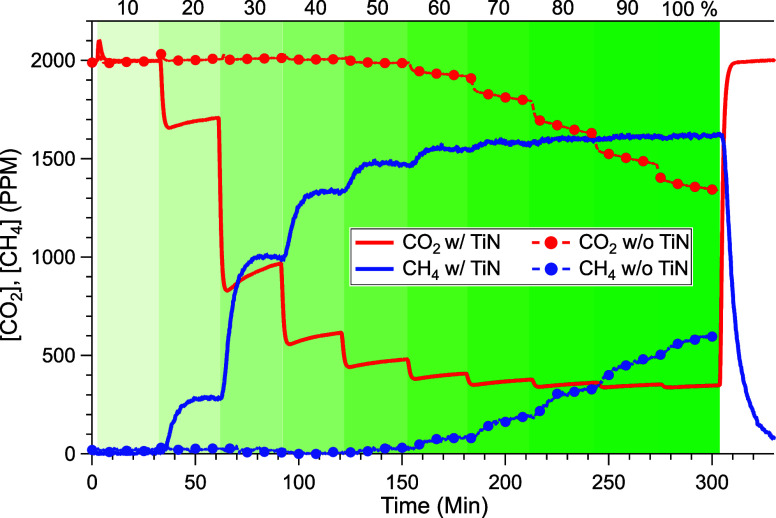
Steady-state hydrocarbon production as a function of light intensity. CO_2_ concentration (red traces) and hydrocarbon production (blue traces) are shown as a function of green light intensity, varied from 0.3 to 2.6 W/cm^2^ in 10% increments. Solid and dashed lines represent data for 5 wt % Ru NPs on TiO_2_, with and without TiN, respectively. Ten sccm of 2000 ppm of CO_2_, 4% H_2_, balance N_2_.

To evaluate photo-RCC performance in an actual capture system, we synthesized two aminopolymer-based composites employing Ru/TiN/TiO_2_, one loaded nominally with 10 wt % branched polyethylenimine (B-PEI), and the other with 10 wt % linear polyethylenimine (L-PEI) (see SI for synthesis and characterization details). We estimated the practical CO_2_ working capacities of these composites under DAC-relevant conditions by measuring the amount of CO_2_ photodesorbed during 10 min of illumination, following a 30 min adsorption period in a stream of 400 ppm of CO_2_ in N_2_ with 10% relative humidity (RH). Under these conditions, the B-PEI composite exhibited a modest ∼0.9 mmol CO_2_ desorbed/g PEI, while the L-PEI composite exhibited a similar working capacity of ∼0.7 mmol CO_2_ desorbed/g PEI. These values are consistent with previous reports on composites with low weight fractions of aminopolymer loadings.^24,25^ Those reports further suggest that working capacities can be increased more than 3-fold with optimization of surface area, aminopolymer molecular weight, amine loading, and humidity. Notably, it is well established that higher humidity levels can enhance the stoichiometry of amine capture and plasticize the polymer matrix, the latter improving sorption kinetics.^26^ For the purposes of this communication, we chose to report baseline RCC performance values of these materials without further optimization of capacity.

To prepare the Ru catalysts for RCC, both composites were activated in a stream of forming gas at 180 °C for 3 h. Photo-RCC experiments were conducted using the B-PEI composite under conditions designed to simulate realistic RCC operation. Following a 15 min CO_2_ loading step, a 30 min forming gas purge was employed to replicate the atmospheric transition necessary for oxygen removal in RCC systems operating with dilute CO_2_ streams. Although the 30 min purge is intentionally excessive and results in some CO_2_ slip, it was used here to unambiguously demonstrate RCC activity. This approach ensures that any observed methane originates from captured CO_2_, rather than from CO_2_ continuously present in the gas stream, as would be the case in a steady-state experiment.

Under completely anhydrous conditions, no methane formation was observed. This result is consistent with the significant reduction in CO_2_ uptake under dry conditions (Figure S13). In the absence of humidity, the B-PEI composite exhibits only a 2-fold increase in CO_2_ uptake relative to the bare TiO_2_ support. In contrast, under humid conditions, where the polymer is more plasticized, CO_2_ uptake increases by more than 50-fold. When the system was operated at 10% RH under RCC conditions, methane production became detectable. However, in experiments using a 400 ppm of CO_2_ stream, the methane signal hovered near the detection threshold of our instrumentation, limiting the accuracy and reliability of the measurements. To improve signal fidelity and confidence in our results, the CO_2_ concentration was increased to 2000 ppm, which substantially enhanced the reliability of methane detection. As a result, this concentration was used in all subsequent RCC experiments.

In the first photo-RCC cycle with the B-PEI composite, ∼55% of the desorbed carbon was detected as methane, with the remaining ∼45% released as unreacted CO_2_. Over the course of just five photo-RCC cycles, hydrocarbon production declined by approximately 75% (Figure 2a, b). To assess the stability of the amine sorbent over these cycles, CO_2_ photodesorption experiments in the absence of forming gas were conducted before and after the five RCC cycles. These tests revealed no measurable loss in CO_2_ capacity, indicating that the amine functionality remained intact. Interestingly, the first photo-RCC cycle released more total gas than the preceding photodesorption experiment, suggesting enhanced desorption, possibly due to moisture-driven gas evolution. We note that moisture produced during methanation may influence both the thermodynamics and kinetics of CO_2_ capture, consistent with prior studies.^26^

**Figure 2:**
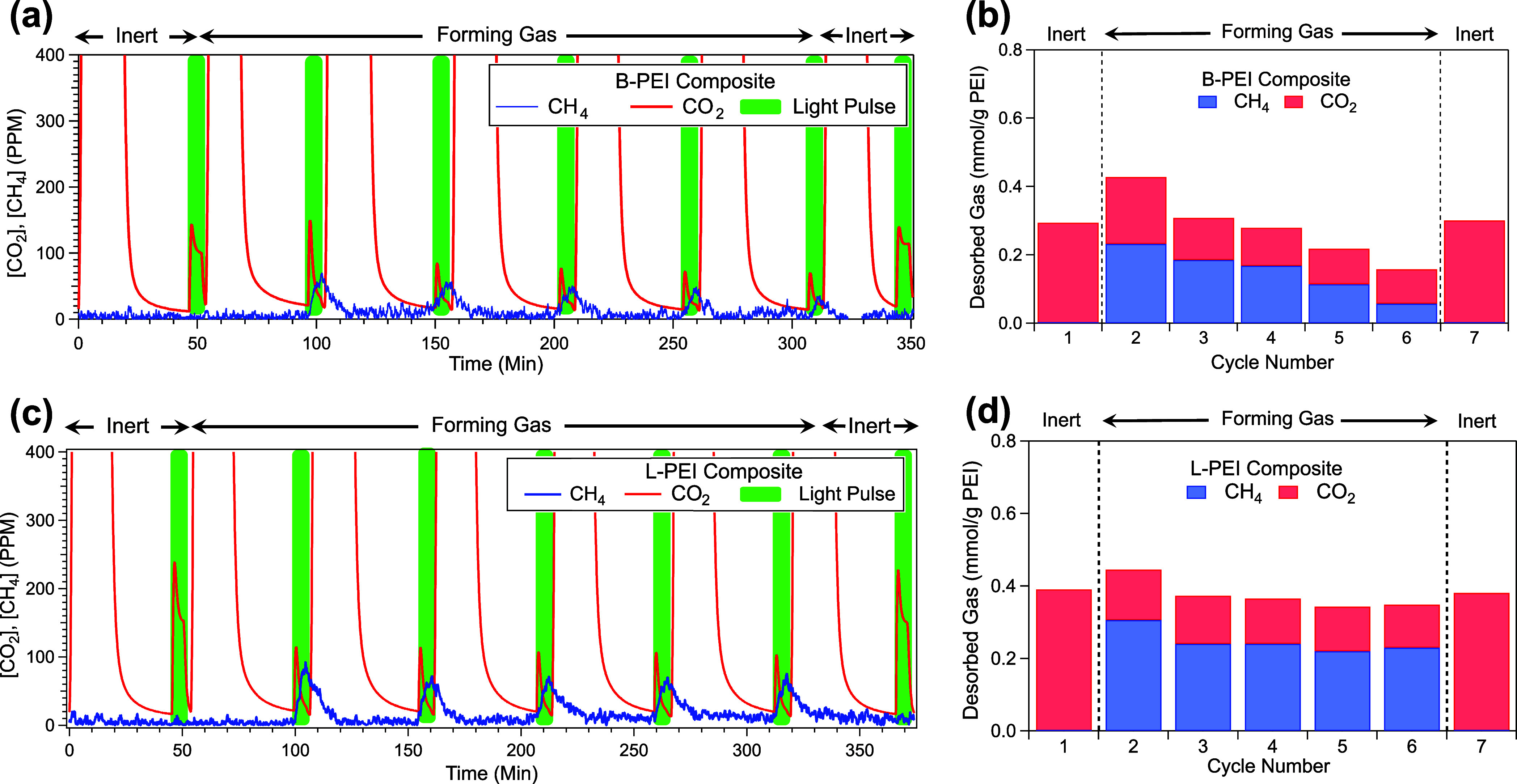
Relative CH_4_ production vs CO_2_ desorption in photo-RCC and photo-desorption cycles. (a, c) Raw LICOR and Enerac data, measuring the concentration of CO_2_ and CH_4_, respectively, for five photo-RCC cycles using a B-PEI composite, Ru/TiN/TiO_2_/B-PEI (a) and L-PEI composite, Ru/TiN/TiO_2_/L-PEI (c) in forming gas, interspersed between two photodesorption cycles in an inert N_2_ environment. (b, d) Relative amounts of CO_2_ and CH_4_ desorbed from B-PEI (b) and L-PEI composites (d). Experimental conditions: Gas flow 10 sccm; Composite mass 11 mg; Adsorption step 2000 ppm of CO_2_, 10% RH, balance N_2_ for 15 min; Purge step 5% RH, balance N_2_ for 30 min (photodesorption cycles), additional 4% H_2_ (RCC cycles); Desorption step 5 min light exposure.

Under the same conditions simulating RCC as employed in the B-PEI system, methane production from the L-PEI system proved to be significantly more stable over five photo-RCC cycles. More than 70% of captured CO_2_ was successfully converted to methane on a given cycle. Additionally, there was no significant loss in CO_2_ capacity of the aminopolymer detected between photodesorption experiments conducted before and after RCC (Figure 2d). Confirmation of complete selectivity of this reaction for methane was obtained through a time-of-flight mass spectrometry (TOF-MS) experiment (Figures S17 and S18) using a modified literature method.^27^

We analyzed the composites with high angle annular dark-field scanning transmission electron microscopy (HAADF-STEM) and elemental composition maps obtained by X-ray energy dispersive spectroscopy (EDS) before and after the RCC experiments. We recorded the Ru NP size distribution in each composite from inspection of >300 individual particles, analyzed using ImageJ software (Figure 3). We note that after the initial 180 °C thermal activation, the Ru NP size distribution in the B-PEI composite was 7.5 ± 2.9 nm, while that of the L-PEI composite was 5.9 ± 2.3 nm, suggesting more significant sintering of Ru NPs had already occurred during the thermal activation in the B-PEI matrix than in the more rigid L-PEI. After the photo-RCC experiments illustrated in Figure 2, the Ru NP size distribution was again measured. Ru NPs in the B-PEI composite had grown slightly to 8.1 ± 3.7 nm, while those in the L-PEI composite remained stable at 5.7 ± 2.5 nm. The increased sintering in the B-PEI composite is consistent with what we know about polymer mobility in these systems, where B-PEI is more flexible and mobile than L-PEI.^28^ This enhanced mobility is a primary reason why B-PEI has been the industry standard for amine-based DAC systems that benefit from rapid sorption kinetics. However, our results suggest that more rigid L-PEI, despite displaying slower desorption kinetics that B-PEI (Figure S11), could help mitigate both CO_2_ slip and NP catalyst sintering in amine-based RCC systems.

**Figure 3:**
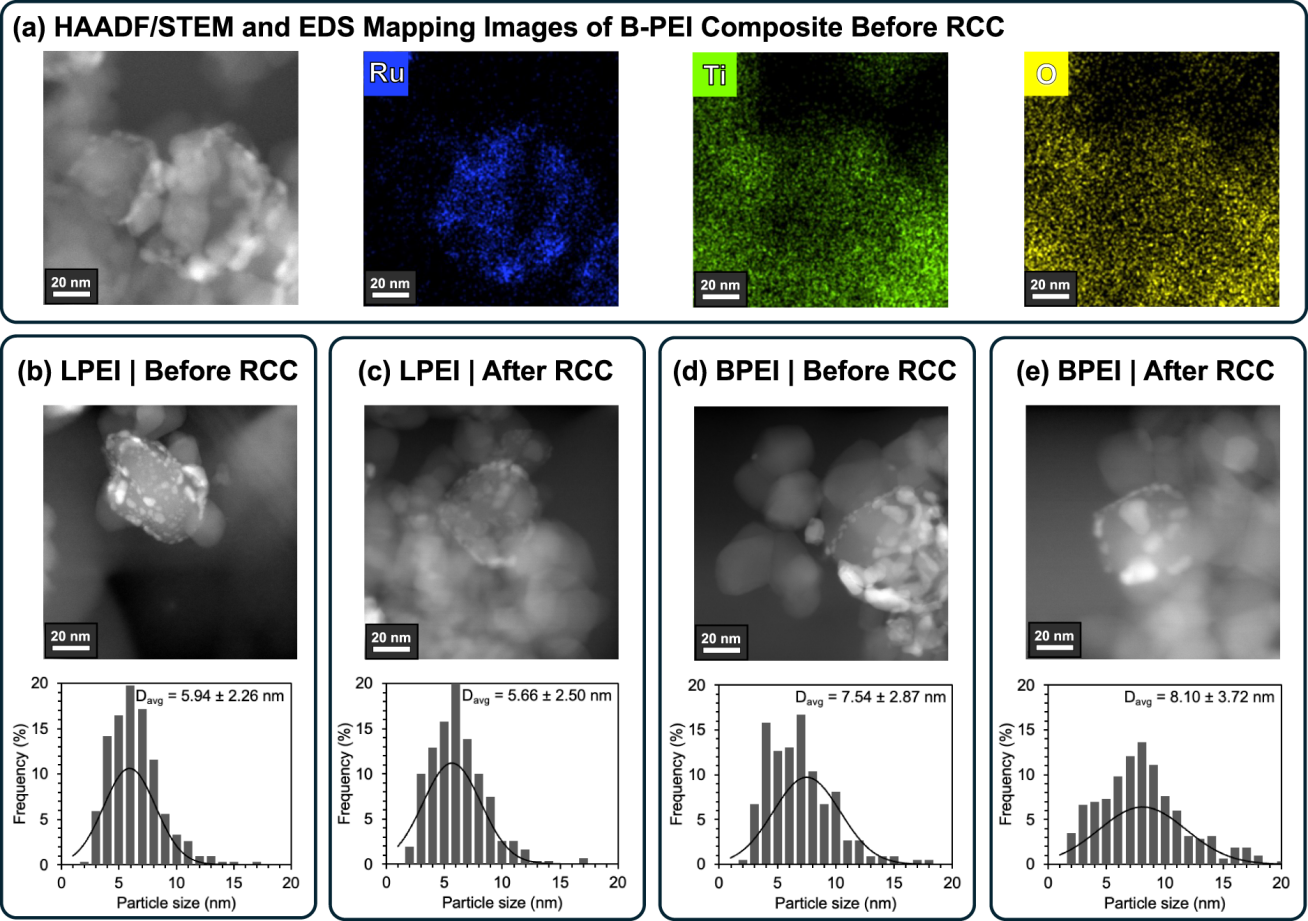
(a) HAADF/STEM and corresponding EDS mapping images of Ru (blue), Ti (green), and O (yellow) for a representative sample of B-PEI composite after thermal treatment of the catalyst but before RCC. (b–e) Ru NP size distributions obtained from inspection of >300 particles of the L-PEI and B-PEI composites, before and after five photo RCC cycles.

Given the observed correlation between polymer matrix mobility and Ru nanoparticle (NP) sintering, we explored an alternative sorbent architecture using small-molecule *N*-propylamines covalently tethered to the surface of TiO_2_ in a Ru/TiN/TiO_2_ composite. We hypothesized that this immobilized amine system would reduce Ru NP sintering by minimizing the mobility of the sorbent matrix. Details of the synthesis, characterization, and preliminary photodesorption and photo-RCC data for this composite are provided in the Supporting Information. In brief, while steady-state photomethanation could be achieved by toggling illumination in a continuous stream of forming gas and 2000 ppm of CO_2_ (Figure S15), methane formation was not observed under RCC conditions, when an N_2_ purge was used prior to illumination to simulate a realistic reactive capture process. Given that the incorporation of N-propylamine into the composite increases CO_2_ uptake relative to the bare support (Figure S13), we attribute this lack of activity in part to rapid CO_2_ slip during the purge phase. Additional experiments revealed that the tethered amines also rapidly lose CO_2_ capacity under irradiation (Figure S16), suggesting the photochemical stability of the surface-bound amine groups may be much lower than the aminopolymer-based systems. Despite efforts to optimize irradiance and minimize purge duration, methane formation under these RCC conditions could not be achieved with the N-propylamine composite in our hands. As a result, we did not prioritize postreaction STEM imaging to assess Ru NP sintering, as the system failed to demonstrate sufficient activity for meaningful comparison. Nevertheless, the results highlight key trade-offs in RCC composite design and underscore the inherent complexity of balancing sorption kinetics, CO_2_ retention, and catalyst stability within a single composite material system.

An early stage technoeconomic viability assessment was performed, intended to evaluate whether this integrated capture-conversion approach could ever be economically viable—even under optimiztic assumptions. In this initial assessment, we incorporated unoptimized experimental values obtained from this study, including a sorbent capacity of 0.4 mmol/g and 70% conversion efficiency of captured CO_2_ to CH_4_. Other parameters, such as the electricity-to-methane conversion efficiency of 50%, were drawn from best-case literature scenarios (see Supporting Information for full assumptions and references) to assess whether the concept could be viable under favorable conditions. Under these assumptions, the estimated methane production cost is ∼$5.19/kg, accounting for installed capital costs, variable operating costs, and system efficiencies—all detailed in the Supporting Information. This cost could be further reduced to $4.98/kg by assuming an 80% conversion efficiency and a sorbent capacity of 2.4 mmol/g, the latter value derived from our recent optimized work on an analogous TiN/TiO_2_/B-PEI composite developed for photo-DAC applications.^29^ Key inputs include an electricity cost of $0.068/kWh and an electrolytic hydrogen cost of $4.50/kg. With access to cheaper electricity of $0.02/kWh and hydrogen sourced at $1/kg, the methane production cost can be decreased to $1.34/kg, within the range of current renewable natural gas production costs ($0.3–1.6/kg).^30^ While these parameters can all be justified, they will require scaling efficiencies to achieve. Nevertheless, they demonstrate the potential economic viability of this system when integrated with renewable energy sources for niche applications and underscore its promise as a sustainable solution for CO_2_ utilization.

There is still much to explore and optimize in this system. Preliminary findings indicate that the Ru catalyst can be activated in forming gas using only a light source, eliminating the need for supplemental heating—a promising advantage for practical field applications. However, a comprehensive study is needed to assess the effects of humidity, the influence of atmospheric oxygen on catalyst stability/regeneration, and the long-term cyclability of these materials. Additionally, the relationship between polymer structure, mobility, and catalyst durability remains an open question. While further refinement is necessary, these results represent one of the more promising demonstrations of an amine-based carbon capture system developed for dilute CO_2_ streams integrated with RCC to date.

## Supplementary Material



## Notes

Data supporting this article: all experimental methods, synthetic procedures, characterization data, additional control experiments, and technoeconomic analyses (PDF)
