# Supplementary material for: Photoreactive Capture and Conversion of Dilute Carbon Dioxide into Synthetic Natural Gas
Source: ACS Appl Energy Mater. 2025 Sep 10;8(18):13179–84. doi: 10.1021/acsaem.5c01559 (PMC12458456; doi:10.1021/acsaem.5c01559)
Supplement: Supplementary file 1 [file ae5c01559_si_001.pdf]

Supporting Information:

Photoreactive Capture and Conversion of Dilute Carbon Dioxide into Synthetic Natural Gas

Sawyer Halingstad,[a] Noemi Leick,[a] Zhe Huang,[a] James M. Crawford,[a,b] Gerard Michael Carroll,[a] Gabrielle A. Kliegle,[a] James L. Young,[a] Alexander J. Hill,[a] Randy Cortright,[a] Matthew M. Yung,[a] and Wade A. Braunecker[a,]*

[a] National Renewable Energy Laboratory, 15013 Denver West Pkwy, Golden, CO 80401

[b] Montana State University, Bozeman, MT 59717, USA

Email: Wade.Braunecker@nrel.gov

Table of Contents

I. Synthetic Procedures……………………..……..……..……………..…………………………S2

II. X-Ray Diffraction (XRD)…………………..………………………………………………...S3

III. Nitrogen Isotherms ……………………...…...…………..……………………………..….S4

IV. Thermogravimetric Analysis (TGA) …………………….…...……………………….….S5

V. Diffuse reflectance infrared Fourier transform spectroscopy (DRIFTS) ………………….…..S6

VI. UV-Vis spectra………..……………….……..………..……………………………….….S7

VII. Light Source...…………………………….……..…………..……………………….…..S7

VIII. Scanning transmission electron microscopy (STEM) ……..……………………..………S8

IX. Thermal Imaging………………….…..…………………..…………………………….…...S9

X. Photo-Desorption and Photo-RCC Experiments....………..…………………….……….…S10

XI. TOF-MS…………………..……………..……..…..……………………………..…………S15

XII. Technoeconomic Analysis (TEA) ………..……………...……………...…………………S17

XIII. References…..……………..…………….....……..……………………………..………S20

I. Synthetic Procedures

Materials. All materials were obtained from Sigma-Aldrich unless otherwise specified. TiO_2 ("P25", Cat. No. 634662) was used as a ~3:1 mixture of anatase and rutile phases. Branched polyethyleneimine (B-PEI) had a number average molecular weight (M_n) of 600 g/mol, while linear PEI (L-PEI) had an M_n of 2500 g/mol. Titanium nitride (TiN) nanoparticles (NPs) were purchased from US Research Nanomaterials, Inc. Previously, we characterized these NPs,[1] where transmission electron microscopy revealed a heterogeneous morphology with an average size distribution centered around ~20 nm. Additionally, diffuse reflectance UV-Vis absorption spectroscopy showed a broad absorption spectrum spanning 380–750 nm.

Preparation of Ru/TiO_2 Catalyst. Incipient wetness impregnation was used to form the 5 wt.% Ru/TiO_2 supports according to a literature procedure.[2] Before methanation experiments, the catalyst was pretreated in a stream of forming gas at 180 °C for ~3 hours (see Section VII. Photo-Desorption and Photo-RCC Experiments, below).

Aminosilane grafting. A modified literature procedure was used for aminosilane grafting.[3] Following calcination of the Ru/TiO_2 catalyst, 200 mg of the dried sample was added to 20 mL of anhydrous toluene. Functionalization was achieved by adding 2.0 mL of 3-aminopropyltriethoxysilane (APTES) to the suspension, stirring at room temperature for 1 hr, and then heating overnight at 80 °C under an inert atmosphere. The product was filtered, washed thoroughly with toluene followed by acetone, and dried on a Schlenk line at ~50 mTorr for 48 hrs at 100 °C. For samples incorporating TiN, 100 mg of the amine-grafted Ru/TiO_2 composite was stirred with 5.3 mg of TiN in methanol for 3 hours. The methanol was removed using a rotary evaporator, and the resulting material was dried under vacuum on a Schlenk line for 48 hours at 100 °C.

Preparation of PEI composites. Nominally ~10 wt.% composites of polyethyleneimine (PEI) were prepared as follows: 21 mg of either branched PEI (B-PEI) or linear PEI (L-PEI) was stirred with 10.5 mg of TiN in 5 mL of methanol for 1 hour. Separately, 180 mg of Ru/TiO_2 was dispersed in methanol and stirred for 1 hour. The two solutions were then combined and stirred for an additional 3 hours. The resulting mixture was subjected to solvent removal using a rotary evaporator. Final degassing was performed on a Schlenk line under vacuum (~100 mTorr) at 110 °C for 48 hours in the dark to eliminate any CO_2, moisture, and residual solvent. The degassed samples were kept under vacuum and transferred directly into an inert atmosphere for subsequent

handling. Thermogravimetric analysis (TGA) burn-off experiments (*vide infra*) determined the final aminopolymer content to be 7.4 wt.% for L-PEI composites and 6.5 wt.% for B-PEI composites.

II. X-Ray Diffraction (XRD)

XRD measurements were conducted on a PANalytical PW3040 X-Ray Diffractometer using Cu Kα radiation (λ = 1.54 Å). The scans were performed at a rate of 2°/min with an operating current of 40 mA and a voltage of 45 kV.

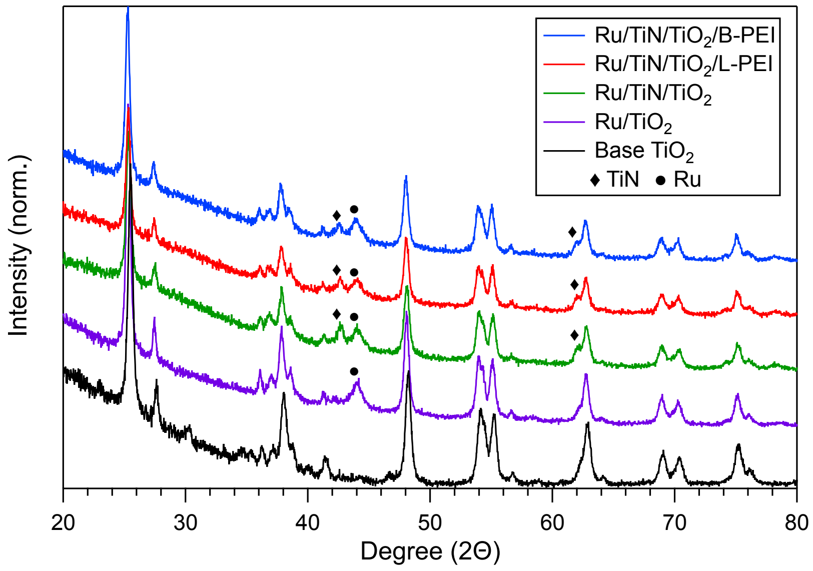

Figure S1. Powder XRD patterns of TiO_2, Ru/TiO_2, Ru/TiN/TiO_2, Ru/TiN/TiO_2/L-PEI, Ru/TiN/TiO_2/B-PEI.

III. Nitrogen Isotherms

The samples underwent degassing under vacuum (~1 × 10^{-5} Torr) at room temperature for 15 hours, followed by a controlled heating ramp to 120 °C over 30 minutes, where they were maintained for an additional 2.5 hours prior to surface area and pore size distribution analysis. This degassing protocol was validated using a custom-built temperature-programmed desorption system equipped with a residual gas analyzer (RGA), which monitored mass-to-charge ratios from 1 to 200 amu, confirming the effective removal of residual solvents and water that could interfere with N_2 adsorption.

N_2 physisorption isotherms at 77 K were collected using a Micromeritics ASAP 2020, employing an equilibration time of 45 seconds for p/p_0 values between 0 and 0.001, which was reduced to 10 seconds for $p/p_0 > 0.001$. The Brunauer-Emmett-Teller (BET) model was applied in the p/p_0 range of 0 to 0.2 while ensuring compliance with the Rouquerol criterion.[4] This analysis yielded specific surface areas of 39.40 ± 0.38 m²/g for the 5 wt.% Ru/TiO_2 sample and 23.80 ± 0.50 m²/g for the 5 wt.% $Ru/TiO_2/TiN$/L-PEI sample.

Pore size distribution and total pore volume were determined using the commercially available Density Functional Theory (DFT) model for cylindrical pore geometries, specifically the "N_2 @77K – oxide cylindrical pores, Strong potential" model within the Micromeritics software, applied in the p/p_0 range of 0.1 to 0.9. Compared to the 5wt% Ru/TiO_2, the pores of width between 120 and 200 Å the 5wt% $Ru/TiO_2/TiN$/L-PEI sample cannot be observed, likely filled with L-PEI.

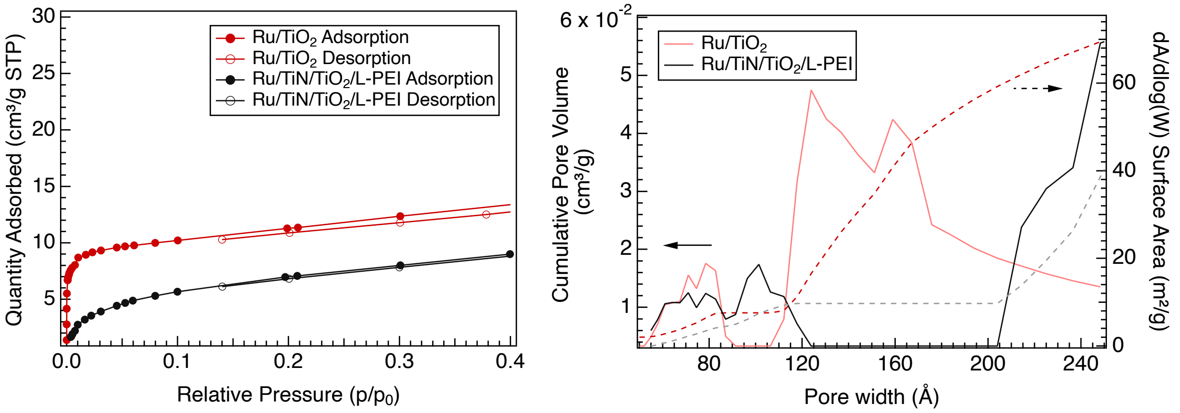

Figure S2. N_2 physisorption isotherms at 77 K for the 5wt% Ru/TiO_2 and 5wt% $Ru/TiN/TiO_2$/L-PEI samples in a) for $p/p_0 \leq 0.4$, and the pore size distribution extracted from the isotherms and applying a DFT-based model in b).

IV. Thermogravimetric Analysis (TGA).

The PEI content in the TiO_2 composites was quantified using a modified TGA method based on a recently reported approach.[5] Measurements were performed on a Setaram SETSYS Evolution under a 100 mL/min flow of zero air. To ensure accurate quantification, the sample was first held at 110 °C to remove adsorbed CO_2 and moisture, then rapidly heated to 250 °C and maintained at that temperature for 8 hours, at which point the mass stabilized. The mass loss observed during this final step was used to estimate the initial PEI content in the composites. Higher burn-off temperatures were avoided, as oxidation reactions involving the Ru NPs introduced complexities that interfered with accurate PEI quantification.

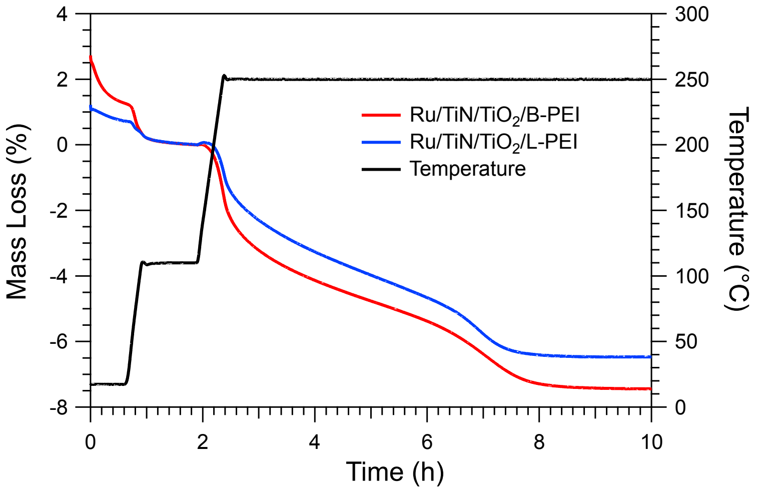

Figure S3. TGA traces of the B-PEI composite (red) and L-PEI composite (blue) held at 250 °C for 8 hours indicate PEI contents of approximately 7.4 wt% and 6.5 wt%, respectively.

V. Diffuse reflectance infrared Fourier transform spectroscopy (DRIFTS)

To verify the presence of the small molecule tethered amine or the aminopolymer in the photocatalyst system, functional groups were measured using DRIFTS. The most differentiating region was the -CH_x stretching bands (2850 – 3000 cm^{-1}), depicted in Fig. S4 for the three material systems considered in this study. All three systems show -CH_x signatures characteristic of L-PEI,[6, 7] B-PEI,[6, 8] and N-propylamine (C_3-NH_2) ,[9, 10] as summarized in Table S1.

Table S1. Assignment of IR peaks/bands observed in DRIFT spectra.

This work	Previous work
**L-PEI**	
2933 cm^{-1}	2930, 2915 cm^{-1}
2891 cm^{-1}	2881, 2899 cm^{-1}
2820 cm^{-1}	2820 cm^{-1}
**B-PEI**	
2949 cm^{-1}	2945, 2946 cm^{-1}
2820 cm^{-1}	2829, 2848 cm^{-1}
**C_3-NH_2**	
2925 cm^{-1}	2925, 2932 cm^{-1}
2860 cm^{-1}	2876, 2823 cm^{-1}

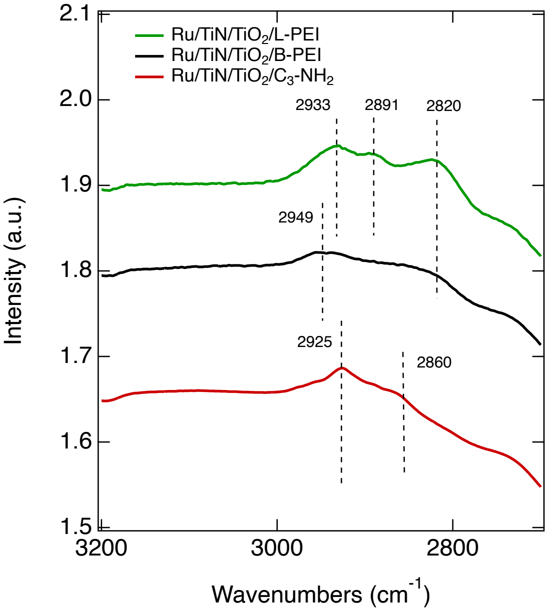

Figure S4. DRIFT spectra in the -CH_x stretching region, for the L-PEI (black), B-PEI (green), and propylamine (red) composite systems. The spectra are offset for clarity, and the peak assignment is summarized in Table S1.

VI. UV-Vis spectra

Diffuse reflectance measurements of the composites were collected on a Cary 5000 UV-Vis spectrometer affixed with an external diffuse reflectance accessory. $BaSO_4$ was used as a non-absorbing standard to calculate the absolute diffuse reflectance at each wavelength for the samples as a function of reflectance. The data are transformed with the equation $F(R) = (1-R)^2/2R$. The samples used in this measurement were densely packed and approximated as an infinitely thick scattering cross section.

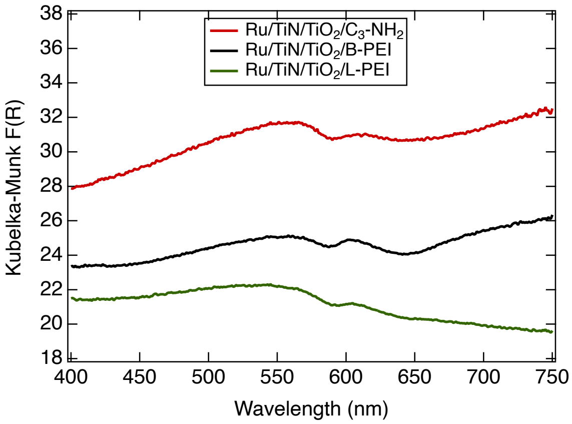

Figure S5. Diffuse reflectance spectra of amine-loaded composites of $Ru/TiN/TiO_2$ over the wavelength range of 400-750 nm.

VII. Light Source

A Prizmatix Optogenetics-LED-Lime-Green module was employed for all photocatalytic experiments. This LED source can provide irradiance up to 470 mW/mm² at the fiber optic cannula tip. The emission spectrum is illustrated in Fig. S6.

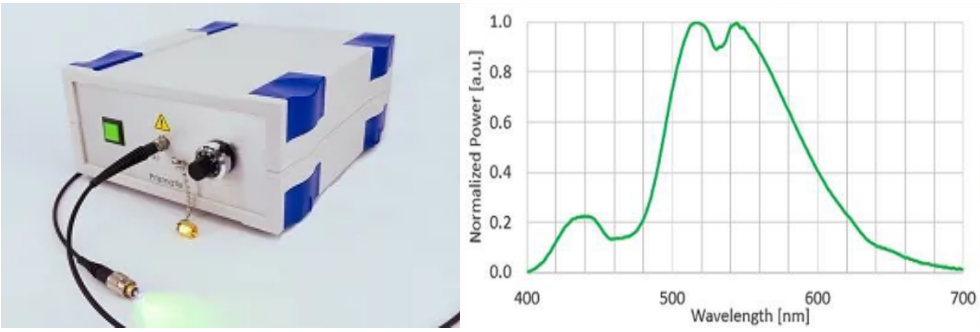

Figure S6. Prizmatix Lime-green high-power LED. Maximum irradiance 470 mW/mm^2.
https://www.prizmatix.com/Optogenetics/optogenetics-led-Lime-Green.htm

VIII. Scanning transmission electron microscopy (STEM)

High angle annular dark-field scanning transmission electron microscopy (HAADF-STEM) and elemental composition maps obtained by x-ray energy dispersive spectroscopy (EDS) were collected using a Spectra200 (ThermoFisher) STEM operating at 200kV in STEM mode with a dwell time of 20-50 µs for EDS map acquisition. Particle size distributions were obtained from inspection of at least 300 particles, analyzed using the ImageJ software.

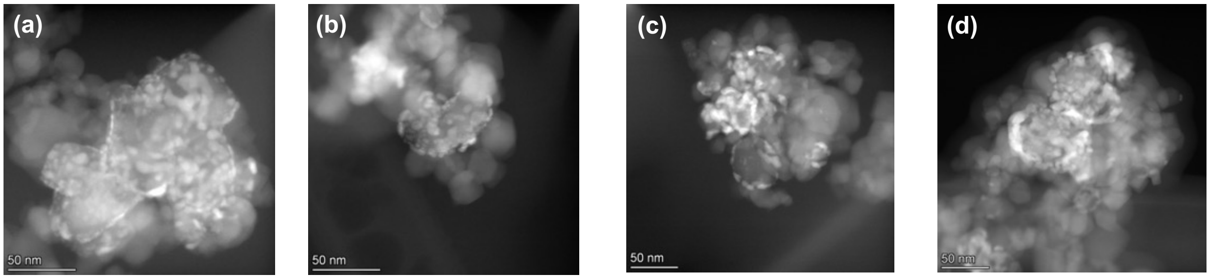

Figure S7. (Top) HAADF/STEM images of (a) fresh Ru/TiN/TiO_2/L-PEI composite, (b) Ru/TiN/TiO_2/L-PEI after photo-RCC cycling, (c) fresh Ru/TiN/TiO_2/B-PEI composite, and (d) Ru/TiN/TiO_2/B-PEI after photo-RCC cycling. (Bottom) Corresponding Ru NP size distributions, determined by analysis of over 300 particles for each of the L-PEI and B-PEI composites.

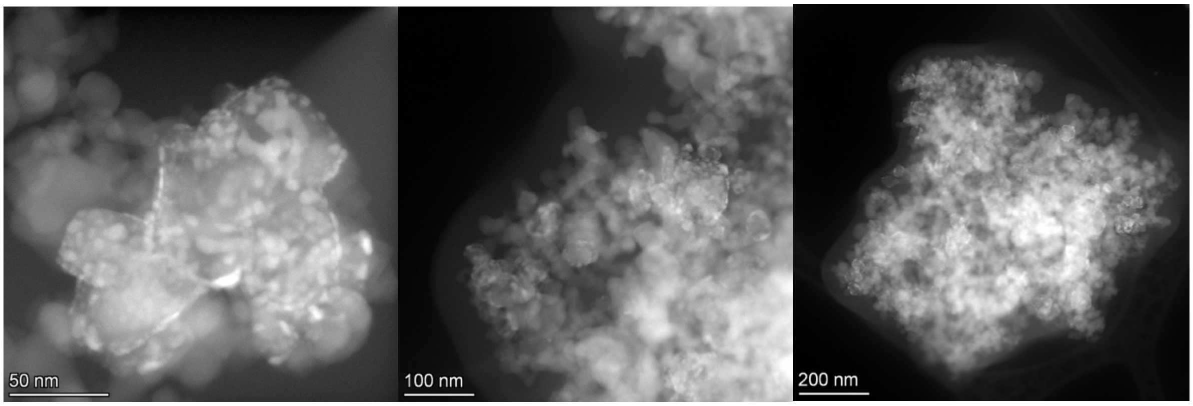

Figure S8. HAADF/STEM images of Ru/TiN/TiO_2/L-PEI composite at different magnitudes.

IX. Thermal Imaging

A FLIR E6-XT series thermal imaging camera was used for this study, featuring a temperature range of -20 to 550 °C, a thermal sensitivity of 60 mK, a frame rate of 9 Hz, and a temperature accuracy of ±2 °C. Measurements were conducted within a dry N_2-filled enclosure to prevent environmental interference. The emissivity of the $TiN/Ru/TiO_2$ samples were estimated at 0.90, calibrated using matte black electrical tape with a known emissivity of 0.95.

A Thorlabs PM160T power meter, equipped with a 9.5 mm sensor aperture, was used to measure optical power at 530 nm. For the experiment, 10 mg of a $TiN/Ru/TiO_2$ composite was illuminated from a distance of 1 cm, replicating the focal length employed during photo-RCC experiments (Fig. S9, top). The surface temperature of the sample under illumination was recorded using the thermal imaging camera (Fig. S9, bottom) and is illustrated in Fig. S10 as a function of varying light intensity. The corresponding optical power at these intensities was simultaneously measured using the power meter and is also plotted in Fig. S10.

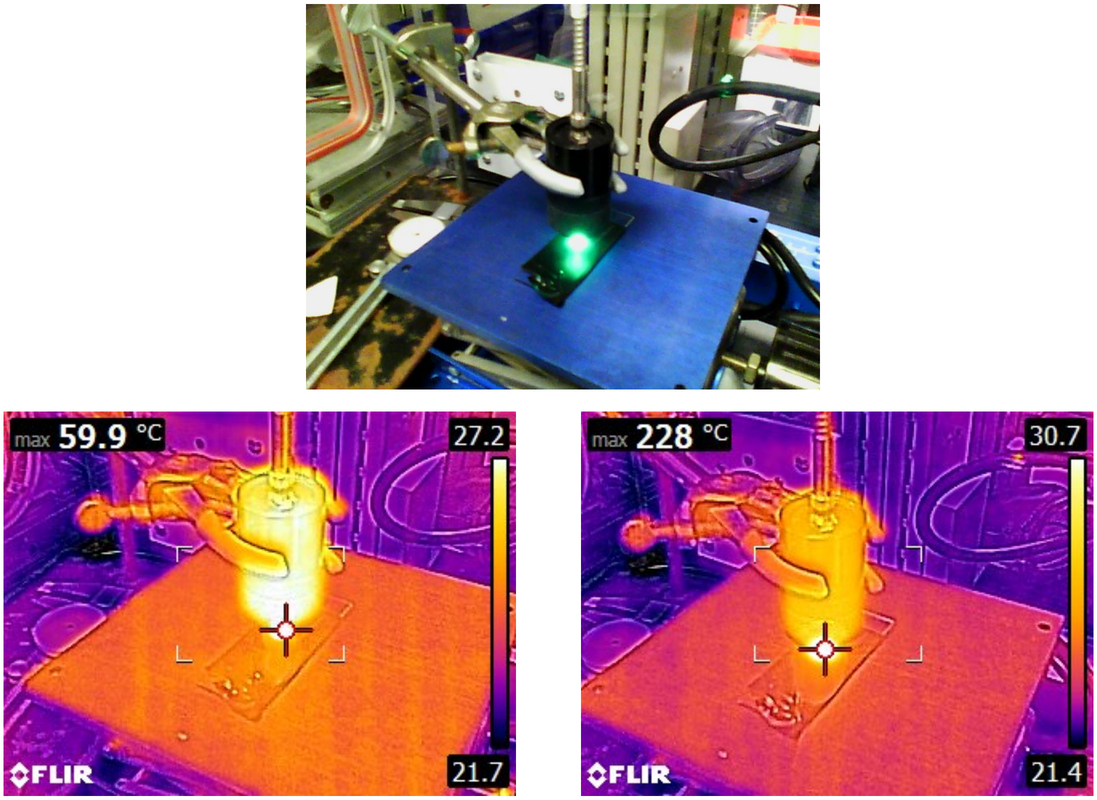

Figure S9. (Top) Irradiation of the $TiN/Ru/TiO_2$ composite in an inert N_2 atmosphere using a Prizmatix lime-green LED equipped with a collimating lens. (Bottom) Sample temperature recorded at 10% light intensity (left) and 100% light intensity (right).

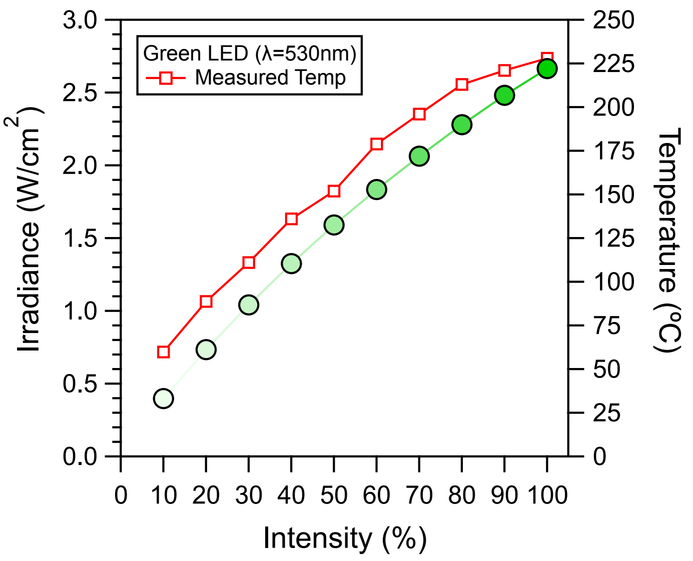

Figure S10. Temperature of the TiN/Ru/TiO_2 composite and corresponding irradiance recorded at 530 nm as a function of relative LED intensity. The LED was focused to a 6 mm spot size.

X. Photo-desorption and photo-RCC experiments.

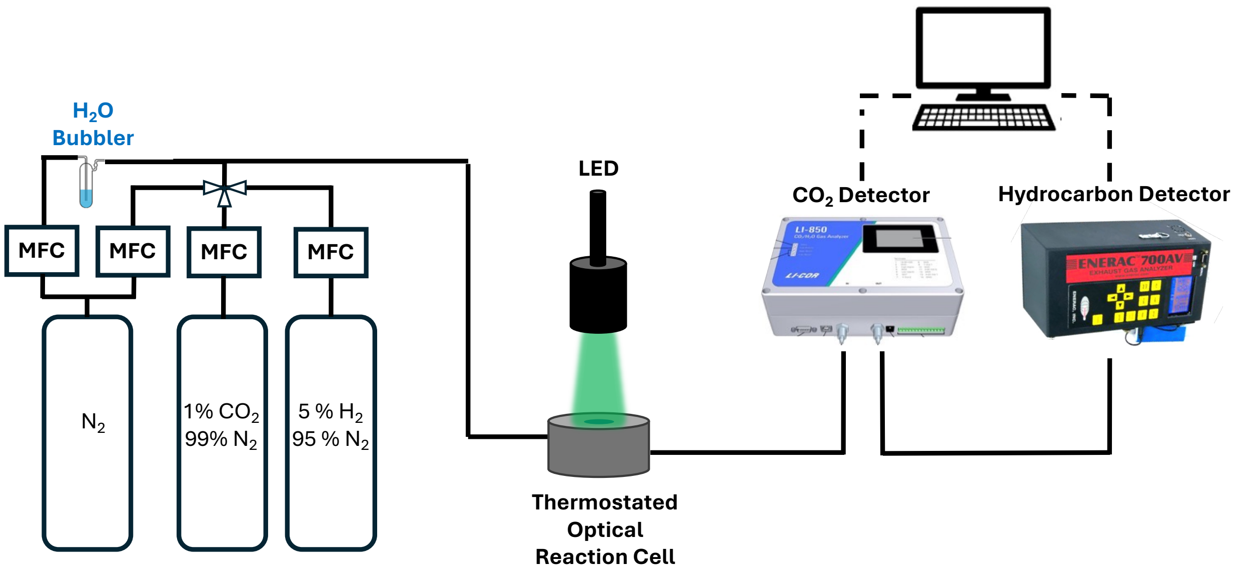

Scheme S1. Flow diagram of photo-reactor setup and detectors.

A. Reactor setup. All catalytic studies were conducted in a Linkham optical stage CCR1000 catalysis cell reactor fitted with temperature control between ambient and 1000 °C. Powder composite samples (10 mg) were mounted on disposable ceramic fabric filters and placed inside a ceramic heating element. The sample was illuminated from above with a Prizmatix Optogenetics Lime-Green LED. Alicat Mass flow controllers were used to feed and mix compressed gases at designated flowrates. The gas composition was analyzed in a LiCOR-850 at STP conditions which continually records ppm-levels of CO_2 and ppt levels of H_2O, in series with an Enerac 700, which continually records ppm-levels of CH_4 and percentages of CO and O_2.

B. Typical Photo-RCC experiment. A total of 10 mg of the catalyst/composite was loaded into the optical stage reaction cell. The Ru catalyst was then activated by flowing forming gas (5% H_2, 95% N_2) at 180 °C for ~3 hrs. CO_2 capture was achieved by introducing a gas stream of 2 sccm 1% CO_2 in N_2 diluted with 8 sccm pure N_2 (~2000 ppm CO_2) for 15 minutes. Following this adsorption step, unbound CO_2 was purged from the chamber headspace by flowing either 10 sccm of pure N_2 (for a photo-desorption experiment) or 10 sccm of forming gas (for photo-RCC) for 30 minutes. A green LED was switched on for 5 minutes, delivering an irradiance of up to ~2.6 W/cm^2 to a 6 mm diameter spot. In a pure N_2 environment (Fig. S13), rapid CO_2 desorption began to occur almost immediately upon light exposure. When forming gas was employed (e.g., Fig. S14), up to 70% of the desorbed carbon was measured as a hydrocarbon by the Enerac detector, confirming successful photo-RCC under these conditions.

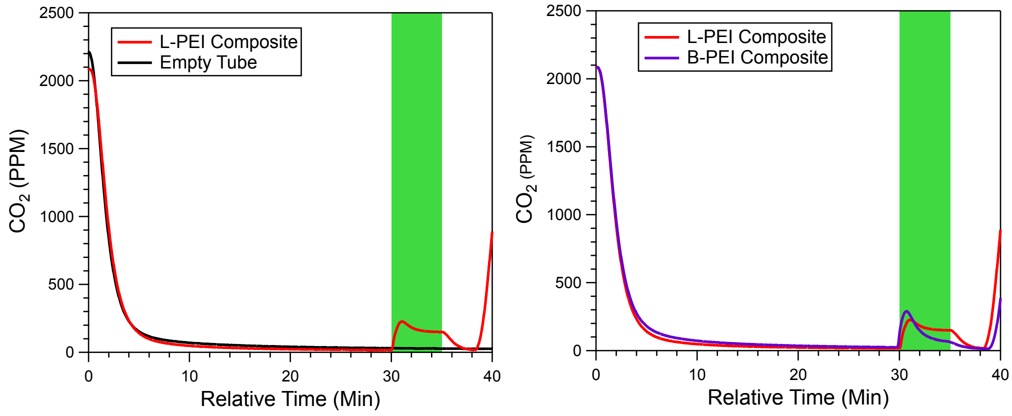

Figure S11. Photo-desorption control experiments. Following CO_2 loading onto the Ru/TiN/TiO_2/PEI composite in a 2000 ppm CO_2 stream (10 sccm total), the headspace was purged

with 10 sccm of pure N_2 for 30 minutes. Subsequently, a green LED was used to illuminate the sample for 5 minutes to induce photo-desorption. (Left) A blank control experiment (black trace) was conducted without a sorbent to confirm background effects and compared to that of the L-PEI composite (red trace). (Right) CO_2 desorption profiles from composites containing L-PEI (red trace) and B-PEI (purple trace), demonstrating the impact of polymer structure on the kinetics of photo-desorption.

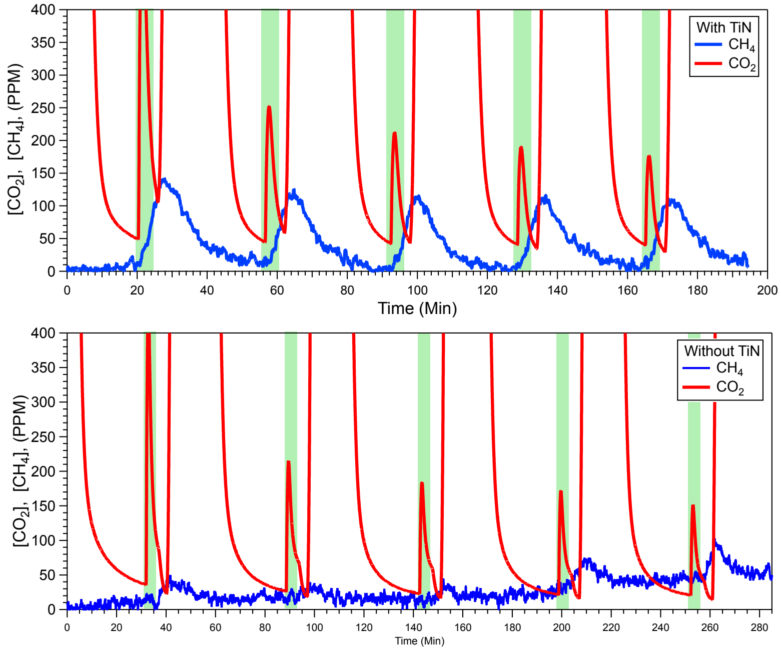

Figure S12. Photo-RCC Cycling with and without TiN. Five cycles of Photo-RCC were performed on the Ru/TiO_2/L-PEI composite under identical conditions (100% LED intensity, 2.6 W/cm^2), with the only variable being the presence of TiN. The top panel shows results with TiN, where significant hydrocarbon production is observed. In contrast, the bottom panel, which lacks TiN, exhibits greatly diminished hydrocarbon formation.

C. Effect of Humidity.

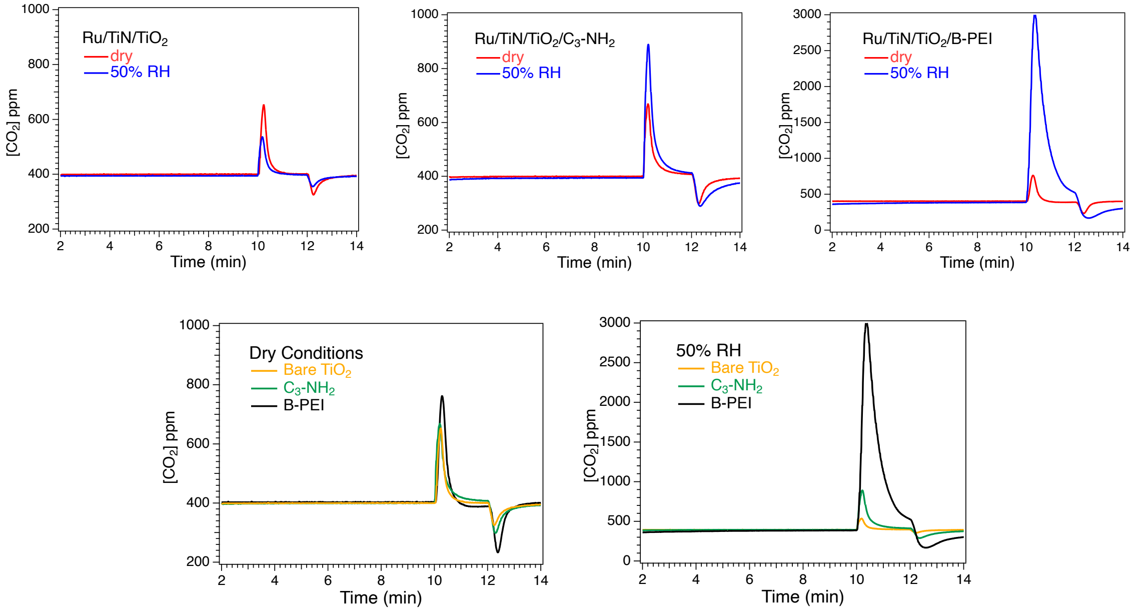

Figure S13. Relative CO_2 desorption as a function of sorbent type and relative humidity. *(Top row)* CO_2 desorption comparison for a single sorbent under dry and humid conditions. *(Bottom row)* CO_2 desorption comparison for a single set of humidity conditions across different sorbents. Experimental conditions: 50 sccm of 400 ppm CO_2, 1.3 W/cm^2 green light irradiation, 10-minute adsorption (dark), followed by 2-minute desorption (light on). Sample mass for each measurement: Ru/TiN/TiO_2 (10 mg), Ru/TiN/TiO_2/C_3-NH_2 (10 mg), Ru/TiN/TiO_2/B-PEI (11 mg).

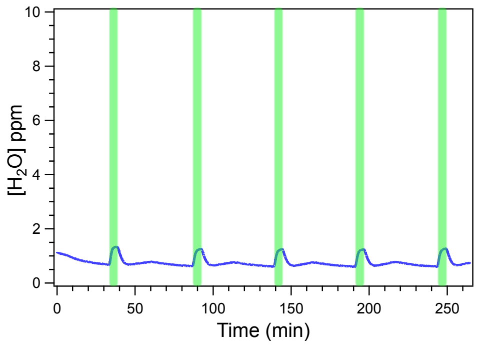

Figure S14. Representative plot of moisture concentration during photo-RCC cycling experiment. A total of 11 mg of Ru/TiN/TiO_2/B-PEI was evaluated in a multi-cycle photo-RCC experiment, as depicted in the main manuscript (Figure 3). Experimental conditions: Gas flow 10 sccm; Adsorption step 2000 ppm CO_2, 10% RH, balance N_2 for 15 min.; Purge step 5% RH, 4% H_2, balance N_2 for 30 min.; Desorption step 5 min. light exposure.

D. Photo-RCC, Grafted Amine.

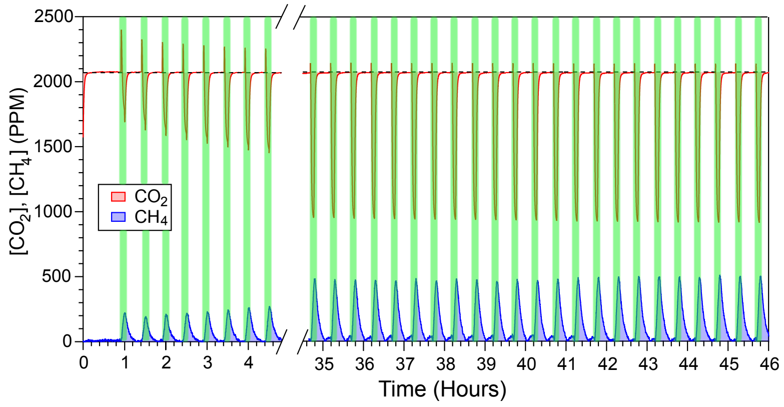

Figure S15. Hydrocarbon Production Under Steady-State Flow with Periodic Light Pulses. Conditions: steady state flow of 2000 ppm CO_2, 4% H_2, balance N_2 over 10 mg of Ru/TiN/TiO_2/C_3-NH_2. A 1.3 W/cm^2 10-minute light pulse (shaded area) was applied after every 20 minutes in the dark to evaluate photocatalytic activity.

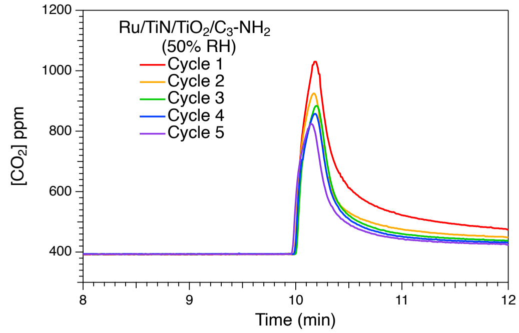

Figure S16. Stability of grafted amine (Ru/TiN/TiO_2/C_3-NH_2) composite. After five cycles, the C_3-NH_2 system exhibits a nearly 50% reduction in CO_2 capacity. Experimental conditions: 50 sccm of 400 ppm CO_2, 1.3 W/cm^2 green light irradiation, 10-minute adsorption (dark), followed by 2-minute desorption (light on).

XI. TOF-MS

The experiments with inline gas analysis via orbital multi-turn time-of-flight mass spectrometer (TOF-MS) utilized a catalyst testbed described in detail elsewhere.[11] The TOF-MS (JEOL, model # JMS-MS3010HRGA) was operated with 24 turns, corresponding to an ion flight path length of approximately 24 m and a mass resolving power of about R = 5000 allowing identification of species by measuring their mass to +/-0.001 amu precision and providing full baseline resolution between isobars separated by as little as 0.005 amu. The measurement configuration parameters were 14 eV ionization energy, 40 μA ion current, 100 °C ion chamber temperature, and 2400 V detector voltage. Gas analysis was performed by a continuous sampling of the cell effluent via 50 μm inner diameter capillary tubing (PEEKsil, Supelco 51332-U, Millipore Sigma) connected to the sample insertion interface of the TOF-MS. The electron ionization energy of 14 eV is significantly lower than the standard electron energy for routine EI mass spectrometry (70 eV). In EI, 70 eV electron energy results in significant fragmentation of chemical species, which is useful for generating fragmentation patterns to identify species, but 70 eV EI lowers the intensity of the parent ion and results in a high density of peaks in the low m/z range. With multi-turn TOF-MS, fragmentation is unnecessary as the species of interest are instead identified by measuring their mass to +/-0.001 amu precision.

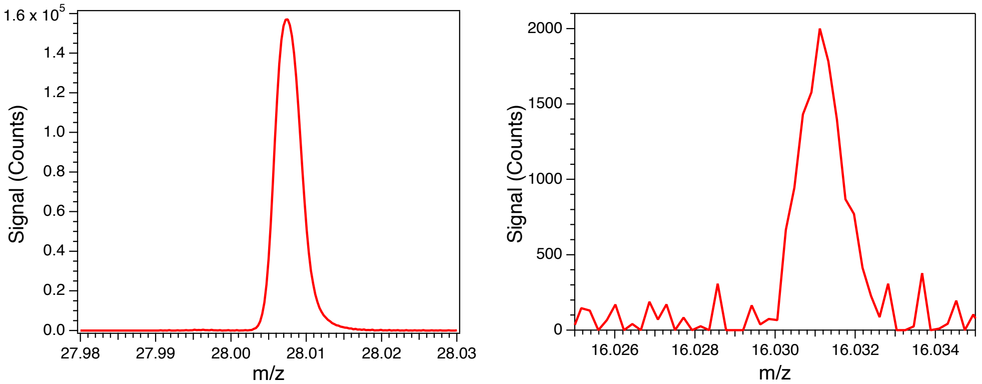

Figure S17. Example TOF-MS spectra of (Left) the region near m/z 28 showing the absence of a CO peak at m/z 27.995 to the left of the N_2 peak at m/z 28.006, and (Right) the CH_4 peak at m/z 16.031.

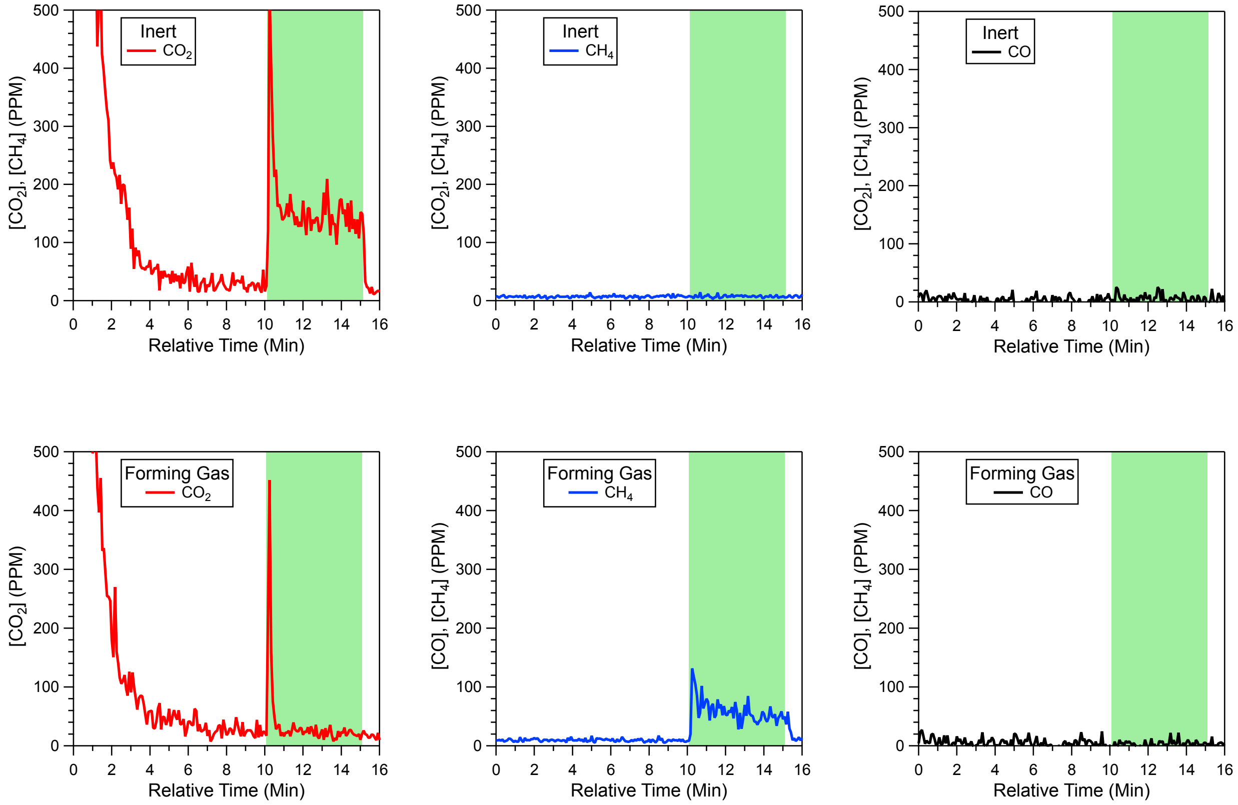

Figure S18. TOF-MS analysis of effluent gas products, tracking mass-to-charge ratios (m/z) 43.990 (CO_2, red), 16.031 (CH_4, blue), and 27.995 (CO, black) for 10 mg of Ru/TiN/TiO_2/L-PEI. (Top row) Photo-desorption experiment: After CO_2 loading in a 2000 ppm CO_2 stream, the chamber was purged with N_2 for 10 min, followed by 5 min of illumination, which induced CO_2 desorption but yielded no detectable CH_4 or CO. (Bottom row) Photo-methanation experiment. After CO_2 loading in a 2000 ppm CO_2 stream, the chamber was purged with 5% H_2 in N_2 for 10 min, followed by 5 min of illumination, which induced some CO_2 desorption but predominantly yielded CH_4.

XII. Technoeconomic analysis (TEA)

The techno-economic analysis for the photo-reactive CO_2 methanation includes four steps: (1) a detailed process flow diagram based on experimental design and data, (2) capital and project cost estimations using in-house models, (3) a discounted cash flow economic model, and (4) the calculation of minimum selling price (MSP). The process flow diagrams include the core CO_2 capture, CO_2 methanation, and product purification stages.

The analysis scale is based on CO_2 flowrate of 1 million metric tons per year, or 126,808 kg per hour, with the plant on stream for 7884 hours per year. With CO_2 concentration of 400 ppm, the input air flowrate is calculated to be 2.09e+8 kg per hour. Based on CO_2 mass flowrate, the materials and energy required for methane production are quantified and used to estimate capital and operating expenses. All costs are adjusted to 2020 United States (US) dollars (2020$) using the Plant Cost Index from Chemical Engineering Magazine, the Industrial Inorganic Chemical Index from SRI Consulting, and the Labor Indices provided by the U.S. Department of Labor Bureau of Labor Statistics. The key process and economic assumptions for TEA are summarized in Table S2. Other standard assumptions for discounted cash flow model and MSP calculation based on our published method[12] are listed in Table S3. Installed capital cost and variable operating cost assumptions are listed in Table S4. Mass and energy inputs are listed in Table S5.

Table S2. Summary of major process and economic assumptions for photo-reactive CO_2 methanation

**Process parameters**	Value	Ref
CO_2 Capture efficiency	80%	[13]
Electricity-to-methane efficiency	50%	[14]
Captured CO_2 conversion to CH_4	70%	This study
Sorbent Capacity	2.4mmol CO_2/g	[15]
H_2/N_2	5%/95%	This study
H_2: Captured CO_2 ratio	4:1	[12, 16]
**Economic parameters**		
State-of-the-art electricity price ($/kWh)	0.068	[12]
State-of-the-art electrolytic hydrogen price ($/kg)	4.5	[12, 17]
Projected future electricity price ($/kWh)	0.02	[17]
Projected future electrolytic hydrogen price ($/kg)	1	[17]

Table S3. Discounted cash flow model assumptions for TEA

Economic parameters	Assumed basis
Basis year for analysis	2020
Debt/equity for plant financing	60%/40%
Interest rate and term for debt financing	8%/10 years
Internal rate of return for equity financing	10%
Total income tax rate	21%
Plant life	30 years
Construction period	3 years
Fixed capital expenditure schedule	32% in year 1
	60% in year 2
	8% in year 3
Start-up time	0.5 year
Revenues during startup	50%
Variable costs during startup	75%
Fixed costs during startup	100%
Site development cost	9% of ISBL, total installed cost
Warehouse	1.5% of ISBL
Working capital	5% of fixed capital investment
Indirect costs	% of total direct costs
Prorated expenses	10
Home office and construction fees	20
Field expenses	10
Project contingency	10
Other costs (start-up and permitting)	10
Fixed Operating Cost	Assumed Basis
Plant operating expenses	3% of total equipment cost (TEC)
Maintenance expenses	3% of total install cost (TIC)
Insurance and Local Tax	0.7% of total capital investment (TCI)

Table S4. Capital and operating costs (Scale: 1 million metric ton/y CO_2 flowrate)

**Installed Capital Cost**	**$MM (Millions)**
Reactor	44.5
Liquid/Gas Flash Tank	8.3
Pressure Swing Adsorption for CH_4 Separation	87.2
LEDs	22.3
Others	2.7
**Total**	**165.0**
**Variable Operating Costs (at $0.068/kWh electricity)**	**$MM/y**
Electricity Cost for Light Power	381.6
H_2 Cost	659.4
Fan Power	13.60
Other Utilities	1.30
Catalyst Makeup	4.73
Other Costs	0.02
**Total**	**1060.6**

Table S5. Mass and energy balance (Scale: 1 million metric ton/y CO_2 flowrate)

**Mass Input**	**kg/hr**
CO_2-In (Mass Flow Rate)	126,808
H_2	18,587
**Mass Output**	**kg/hr**
CH_4	25,627
Unconverted CO_2	55,796
Wastewater	57,452
Unconverted H_2	5,576
Vapor	653
CH_4 Waste	290
**Energy Input**	**kW**
CH_4 PSA Electricity	2,403
Air Fan Electricity	25,361
Light Power	711,866
**Total**	**739,640**

XIII. References

1. Leick, N.; Halingstad, S.; Crawford, J. M.; Carroll, G. M.; Yung, M. M.; Cortright, R.; Braunecker, W. A., Photo-swing CO2 capture using a branched polyethylenimine sorbent and TiN light absorber. *J. Mater. Chem. A,* ***2025****, Advance Article*, DOI: 10.1039/d5ta02824h.
2. Crawford, J. M.; Petel, B. E.; Rasmussen, M. J.; Ludwig, T.; Miller, E. M.; Halingstad, S.; Akhade, S. A.; Pang, S. H.; Yung, M. M., Influence of residual chlorine on Ru/TiO2 active sites during CO2 methanation. *Appl. Catal. A-Gen.* **2023,** *663*, 119292.
3. Alkhabbaz, M. A.; Bollini, P.; Foo, G. S.; Sievers, C.; Jones, C. W., Important Roles of Enthalpic and Entropic Contributions to CO2 Capture from Simulated Flue Gas and Ambient Air Using Mesoporous Silica Grafted Amines. *J. Am. Chem. Soc.* **2014,** *136* (38), 13170-13173.
4. Rouquerol, J.; Llewellyn, P.; Rouquerol, F., Is the bet equation applicable to microporous adsorbents? In *Studies in Surface Science and Catalysis*, Llewellyn, P. L.; Rodriquez-Reinoso, F.; Rouqerol, J.; Seaton, N., Eds. Elsevier: 2007; Vol. 160, pp 49-56.
5. Potter, M. E.; Pang, S. H.; Jones, C. W., Adsorption Microcalorimetry of CO2 in Confined Aminopolymers. *Langmuir* **2017,** *33* (1), 117-124.
6. Grenda, K.; Idström, A.; Evenäs, L.; Persson, M.; Holmberg, K.; Bordes, R., An analytical approach to elucidate the architecture of polyethyleneimines. *J. Appl. Polym. Sci.* **2022,** *139* (7), 51657.
7. Lakard, S.; Herlem, G.; Lakard, B.; Fahys, B., Theoretical study of the vibrational spectra of polyethylenimine and polypropylenimine. *J. Mol. Struct. THEOCHEM* **2004,** *685* (1), 83-87.
8. Russell-Parks, G. A.; Leick, N.; Marple, M. A. T.; Strange, N. A.; Trewyn, B. G.; Pang, S. H.; Braunecker, W. A., Fundamental Insight into Humid CO2 Uptake in Direct Air Capture Nanocomposites Using Fluorescence and Portable NMR Relaxometry. *J. Phys. Chem. C.* **2023,** *127* (31), 15363-15374.
9. https://www.chemicalbook.com/SpectrumEN_107-10-8_IR1.htm.
10. https://www.chemicalbook.com/SpectrumEN_107-10-8_IR2.htm.
11. Wilder, L. M.; Balogun, K.; Klein, W. E.; Chumble, P.; Young, J. L., Nitrogen Reduction Testing with Real-Time 15NH3 Yield Quantification Using Orbital Multiturn Time-of-Flight Mass Spectrometry. *ACS Energy Lett.* **2024,** *9* (12), 5780-5786.

12. Huang, Z.; Grim, R. G.; Schaidle, J. A.; Tao, L., The economic outlook for converting CO2 and electrons to molecules. *Energy Environ. Sci.* **2021,** *14* (7), 3664-3678.

13. Sodiq, A.; Abdullatif, Y.; Aissa, B.; Ostovar, A.; Nassar, N.; El-Naas, M.; Amhamed, A., A review on progress made in direct air capture of CO2. *Environ. Technol. Innov.* **2023,** *29*, 102991.

14. Sastre, F.; Versluis, C.; Meulendijks, N.; Rodríguez-Fernández, J.; Sweelssen, J.; Elen, K.; Van Bael, M. K.; den Hartog, T.; Verheijen, M. A.; Buskens, P., Sunlight-Fueled, Low-Temperature Ru-Catalyzed Conversion of CO2 and H2 to CH4 with a High Photon-to-Methane Efficiency. *ACS Omega* **2019,** *4* (4), 7369-7377.

15. Varghese, A. M.; Karanikolos, G. N., CO2 capture adsorbents functionalized by amine – bearing polymers: A review. *Int. J. Greenhouse Gas Control* **2020,** *96*, 103005.

16. Jaffar, M. M.; Nahil, M. A.; Williams, P. T., Parametric Study of CO2 Methanation for Synthetic Natural Gas Production. *Energy Technol.* **2019,** *7* (11), 1900795.

17. NREL, H2A: Hydrogen Analysis Production Models, https://www.nrel.gov/hydrogen/h2a-production-models.html (accessed 8/2025).
